# Uterine artery embolization in Tanzania: a procedure with major public health implications

**DOI:** 10.1186/s42155-023-00384-9

**Published:** 2023-08-07

**Authors:** Balowa Musa, Jared Mark Alswang, Rose Di Ioia, Lydia Grubic, Azza Naif, Erick Michael Mbuguje, Victoria Vuong, Janice Newsome, Behnam Shaygi, Vijay Ramalingam, Fabian Max Laage Gaupp

**Affiliations:** 1 Radiology and Imaging Department, School of Medicine, Muhimbili University of Health and Allied Sciences, P.O. Box 65001, Dar Es Salaam, Tanzania; 2 Harvard Medical School, Harvard University, 25 Shattuck Street, Boston, MA 02115 USA; 3 Faculty of Medicine and Health Sciences, McGill University, 2001 McGill College Avenue, Montreal, QC H3A 1A3 Canada; 4 Marian University College of Osteopathic Medicine, 3200 Cold Spring Rd, Indianapolis, IN 46222 USA; 5 Department of Radiology and Imaging, Muhimbili National Hospital, P.O. Box 65000, Dar Es Salaam, Tanzania; 6 Department of Radiology, University of California San Diego Medical Center, 200 W. Arbor Drive, San Diego, CA 92103 USA; 7 Department of Radiology, Emory University School of Medicine, 1364 Clifton Rd, NE, Atlanta, GA 30322 USA; 8 Department of Radiology, London North West University Healthcare NHS Trust, A404 Watford Rd, Harrow, HA1 3UJ UK; 9 Division of Interventional Radiology, Department of Radiology, Beth Israel Deaconess Medical Center, Harvard Medical School, 1 Deaconess Road, Boston, MA 02215 USA; 10 Section of Interventional Radiology, Department of Radiology and Biomedical Imaging, Yale School of Medicine, 333 Cedar St, New Haven, CT 06510 USA

**Keywords:** Uterine artery embolization, Uterine fibroids, Adenomyosis, Postpartum hemorrhage, Interventional radiology, Uterine fibroid embolization, Reproductive health, Sub-Saharan Africa, Tanzania

## Abstract

**Background:**

The burden of uterine fibroids is substantial in sub-Saharan Africa (SSA), with up to 80% of black women harboring them in their lifetime. While uterine artery embolization (UAE) has emerged as an effective alternative to surgery to manage this condition, the procedure is not available to the vast majority of women living in SSA due to limited access to interventional radiology (IR) in the region. One of the few countries in SSA now offering UAE in a public hospital setting is Tanzania. This study aims to assess the safety and effectiveness of UAE in this new environment.

**Methods:**

From June 2019 to July 2022, a single-center, retrospective cohort study was conducted at Tanzania’s first IR service on all patients who underwent UAE for the management of symptomatic fibroids or adenomyosis. Patients were selected for the procedure based on symptom severity, imaging findings, and medical management failure. Procedural technical success and adverse events were recorded for all UAEs. Self-reported symptom severity and volumetric response on imaging were compared between baseline and six-months post-procedure using paired sample t-tests.

**Results:**

During the study period, 92.1% (*n* = 35/38) of patients underwent UAE for the management of symptomatic fibroids and 7.9% (*n* = 3/38) for adenomyosis. All (*n* = 38/38) were considered technically successful and one minor adverse event occurred (2.7%). Self-reported symptom-severity scores at six-months post-procedure decreased in all categories: abnormal uterine bleeding from 8.8 to 3.1 (-5.7), pain from 6.7 to 3.2 (-3.5), and bulk symptoms from 2.8 to 1 (-1.8) (*p* < 0.01). 100% of patients reported satisfaction with outcomes. Among the nine patients with follow-up imaging, there was a mean volumetric decrease of 35.5% (*p* = 0.109).

**Conclusions:**

UAE for fibroids and adenomyosis can be performed with high technical success and low complication rates in a low-resource setting like Tanzania, resulting in significant symptom relief for patients. Building capacity for UAE has major public health implications not only for fibroids and adenomyosis, but can help address the region’s leading cause of maternal mortality, postpartum hemorrhage.

## Background

Promoting maternal and reproductive health is a long-standing priority in the field of global health. While impressive strides have been made advancing the field in the last two decades [1–4], potential contributions from various specialties have been under-utilized. Interventional radiology (IR) is a noteworthy example. In high-income countries, IR plays a significant role in maternal and reproductive health with a wide-range of procedures at their disposal to treat conditions ranging from obstructive uropathy from cervical cancer to pelvic congestion syndrome [5]. One such high-yield IR procedure with significant potential to make a global impact in the field is uterine artery embolization (UAE), commonly used to manage uterine fibroids, adenomyosis, and postpartum hemorrhage.

Uterine fibroids or leiomyomas, the most common benign neoplasm in women, are highly endemic within sub-Saharan Africa (SSA) and constitute a considerable burden on its female population [6, 7]. This very common condition can have significant negative impact on patients’ lives with many affected women experiencing menorrhagia, menometrorrhagia, dysmenorrhea, pelvic pain, dyspareunia, infertility, and/or bulk symptoms [8] With stark racial disparities in the incidence of uterine fibroids, and black women far more likely to harbor them, estimated at 70–80% in their lifetime, and be symptomatic, their management in SSA should be considered a public health priority [6, 9].

Unfortunately, when medical management fails, the only available treatment option in most of the region remains to be hysterectomy, with myomectomy only available in a few specialized centers [10, 11]. UAE, a safe and effective alternative to surgery, has been routinely performed for decades in high-income countries [12–14]. This minimally invasive procedure involves the controlled injection of an embolic agent into the uterine arteries under fluoroscopic-guidance by an interventional radiologist, thus restricting its blood flow, leading to a reduction of abnormal uterine bleeding and regression in size and associated symptoms over the following weeks [15].

Until recently, there was no IR training program in East Africa, accounting for low UAE accessibility for its population. This changed in 2018 with the establishment of Tanzania’s first IR service and training program at Muhimbili National Hospital (MNH), Tanzania’s national referral center [16]. Elective UAEs for symptomatic fibroids and adenomyosis have since become readily integrated into clinical practice and emergent UAE for the management of postpartum hemorrhage (PPH), the leading cause of maternal morbidity and mortality in SSA [17], will be offered starting in the summer of 2023 with the opening of MNH’s new angiography suite. This retrospective study assesses the safety and effectiveness of UAE as a first-line non-medical therapy for symptomatic fibroids and adenomyosis at MNH and serves as a proof-of-concept for wider adoption of this procedure in the region as a treatment option for symptomatic uterine fibroids, adenomyosis, and PPH going forward.

## Methods

A single-center, retrospective, observational cohort study was conducted at a large tertiary hospital in Tanzania on all patients who underwent UAE for the management of symptomatic uterine fibroids and adenomyosis from June 2019 to July 2022. Throughout the study period, IR faculty from North America and Europe traveled to Tanzania on a monthly basis to provide hands-on teaching for Tanzanian IR fellows. All UAEs were performed by Tanzanian IR fellows as primary operators under the supervision of local or visiting IR faculty [16].

Ethical clearance (MNH/IRB/EXT/2022/008) was obtained, and all patients provided informed consent for the procedure and use of their data in research. Patients with symptomatic uterine fibroids or adenomyosis not responsive to medical management were considered for UAE. Exclusion criteria for UAE included pregnancy, active uterine infection, malignancy of the uterus, and pedunculated subserosal fibroids [18, 19]. Preprocedural workup included detailed medical history, contrast-enhanced Magnetic Resonance Imaging (MRI), routine pre-procedural hematologic evaluation, urine pregnancy test, and pap smear. UAEs were performed following standard international practice guidelines [20, 21].

Pre-procedural, procedural, and follow-up data were collected and stored in a Research Electronic Data Capture (REDCap) database (REDCap 8.7.1—©2021 Vanderbilt University). Contrast-enhanced pelvic MRI was performed pre-procedure and at six-month follow-up, and the maximum diameter and calculated volume of the dominant fibroid were recorded. Fibroid volume was calculated using the ellipsoid formula (length x width x height) × 0.52 [22]. Adverse events were classified according to the Society of Interventional Radiology (SIR) guidelines [23]. Symptom severity was assessed pre-procedure and six-months post-procedure by telephone interview in the following categories: abnormal uterine bleeding (including irregular menstruation, menometrorrhagia, and menorrhagia), pain (including dysmenorrhea, abdominal pain, back pain, and pelvic pain), and bulk symptoms (including pressure, pelvic fullness, urinary incontinence, bloating, urinary frequency, and constipation), all scored on a 1–10 self-reported Likert-type scale (1 = least severe, 10 = most severe).

Descriptive analysis was performed and numerical variables were summarized by mean and standard deviation and categorical variables by frequency. Paired t-tests were used to compare pre- and post-procedure dominant fibroid size and symptom severity. Statistical analysis was completed using Microsoft Excel 2019 (Microsoft Corporation, Redmond, Washington, United States).

## Results

From June 2019 to July 2022, 38 patients who underwent uterine artery embolizations were included in this study: 92.1% (*n* = 35/38) for symptomatic uterine fibroids and 7.9% (*n* = 3/38) for adenomyosis. The mean age of the study population was 41.6 years (range: 28 to 52 years). Referral data was collected from 89.5% (*n* = 34/38) of patients. The majority of women (*n* = 20/34; 58.8%) presented as referrals from the MNH Obstetrics and Gynecology Department, 38.2% (*n* = 13/34) were self-referred, and 2.9% (*n* = 1/34) were referred by friends and family. Payment information was available from 94.7% (*n* = 36/38): 52.8% (*n* = 19/36) self-paid and 47.2% (*n* = 17/36) had their procedures covered by insurance (Table 1). The mean out-of-pocket costs for uterine artery embolization was $665.28 USD (1,600,000 Tanzanian Shillings) compared to $831.60 USD (2,000,000 Tanzanian Shillings) for hysterectomy. Presenting symptoms within the patient population included abnormal uterine bleeding, bulk symptoms, pain, subfertility, and anemia (Table 2).

**Table 1:** Demographic characteristics of the study population

Variable	Characteristics	Frequency	Percent
		***n* = 37**	
Age	18–29	1	2.7
	30–39	14	37.8
	> 40	22	59.5
		***n* = 36**	
Language	Swahili	33	91.7
	English	3	8.3
		***n* = 35**	
Region	Dar Es Salaam	29	82.9
	Arusha	2	5.7
	Pwani	2	5.7
	Zanzibar Mjini Magharibi	1	2.9
	Mtwara	1	2.9
		***n* = 36**	
Religion	Christian	30	83.3
	Muslim	6	16.6
		***n* = *36***	
Payment method	Self	18	50.0
	Insurance	17	47.2
	Family/friends	1	2.8

**Table 2:** Presenting symptoms and diagnoses of the study population

Variable	Characteristic	Frequency *n* = 38	Percent
Presenting Symptoms	Abnormal uterine bleeding^a^	31	81.6
	Bulk Symptoms^b^	13	34.2
	Pain^c^	13	34.2
	Subfertility^d^	4	10.5
	Anemia	4	10.5
	Uterine fibroids	35	92.1
Principal Diagnosis	Adenomyosis	3	7.9

^a^Includes irregular menstruation, menometrorrhagia, and menorrhagia

^b^Includes pressure, pelvic fullness, urinary incontinence, bloating, urinary frequency, constipation

^c^Includes dysmenorrhea, dysmenorrhagia, abdominal pain, pelvic pain, lower back pain, and pain included in same category

^d^Includes spontaneous miscarriages and infertility

**Fig. 1:**
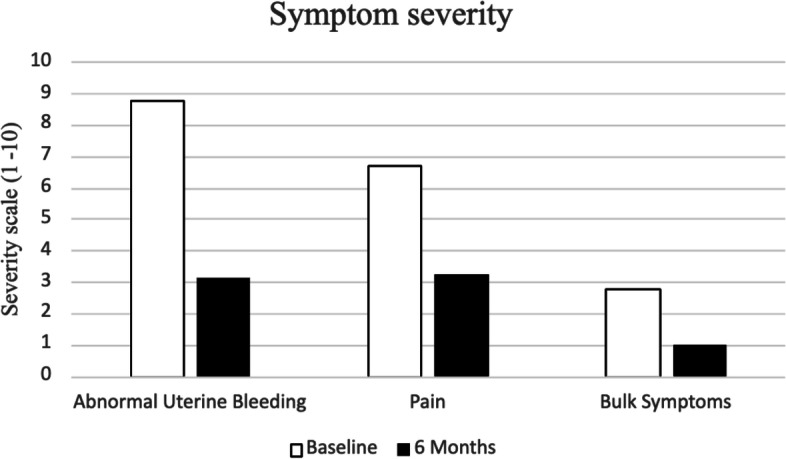
Mean symptom severity score at baseline and 6 months post-procedure

**Fig. 2:**
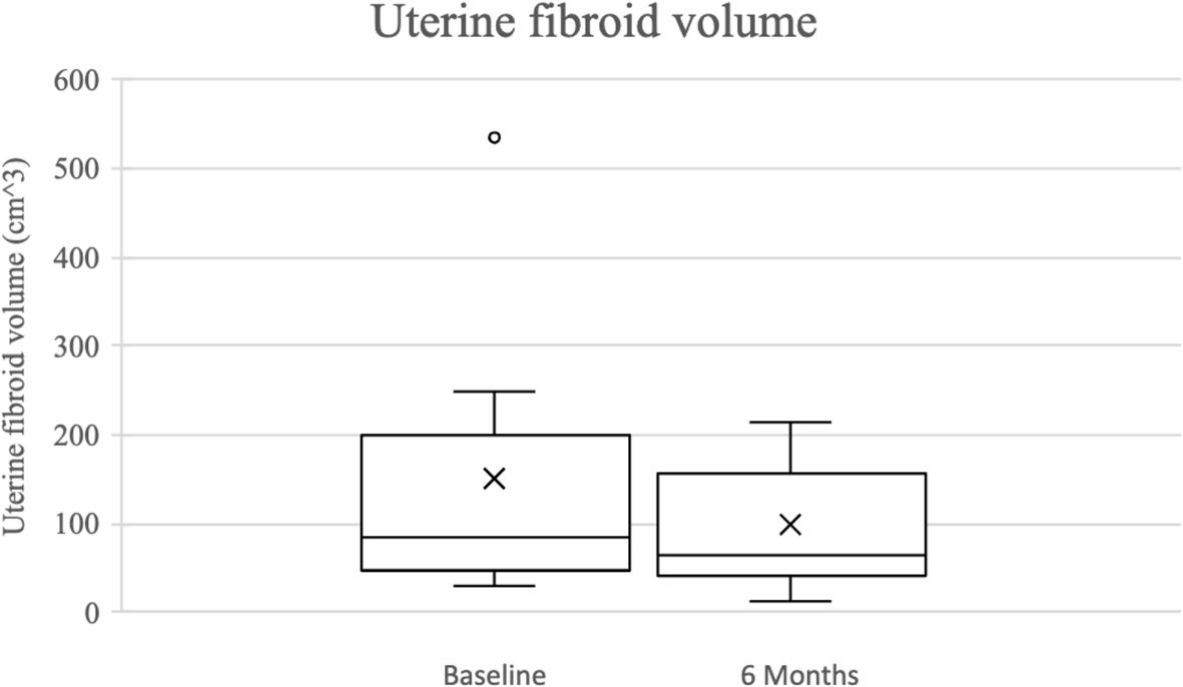
Dominant uterine fibroid volume at baseline and 6 months post-procedure

**Fig. 3:**
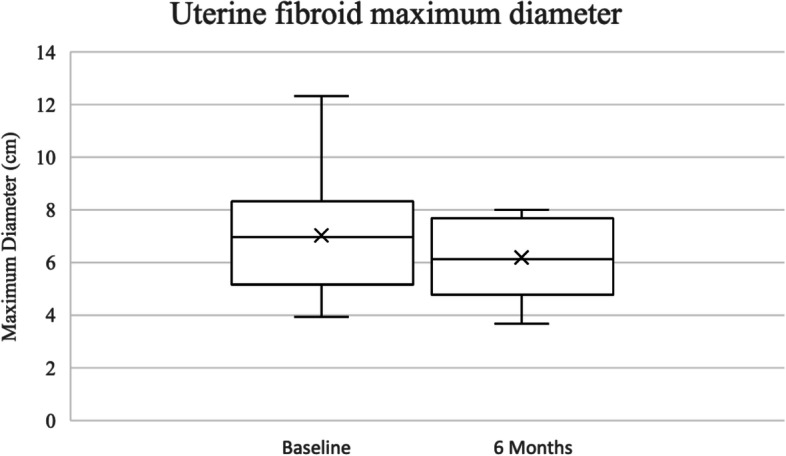
Dominant uterine fibroid maximum diameter at baseline and 6 months post-procedure

**Fig. 4:**
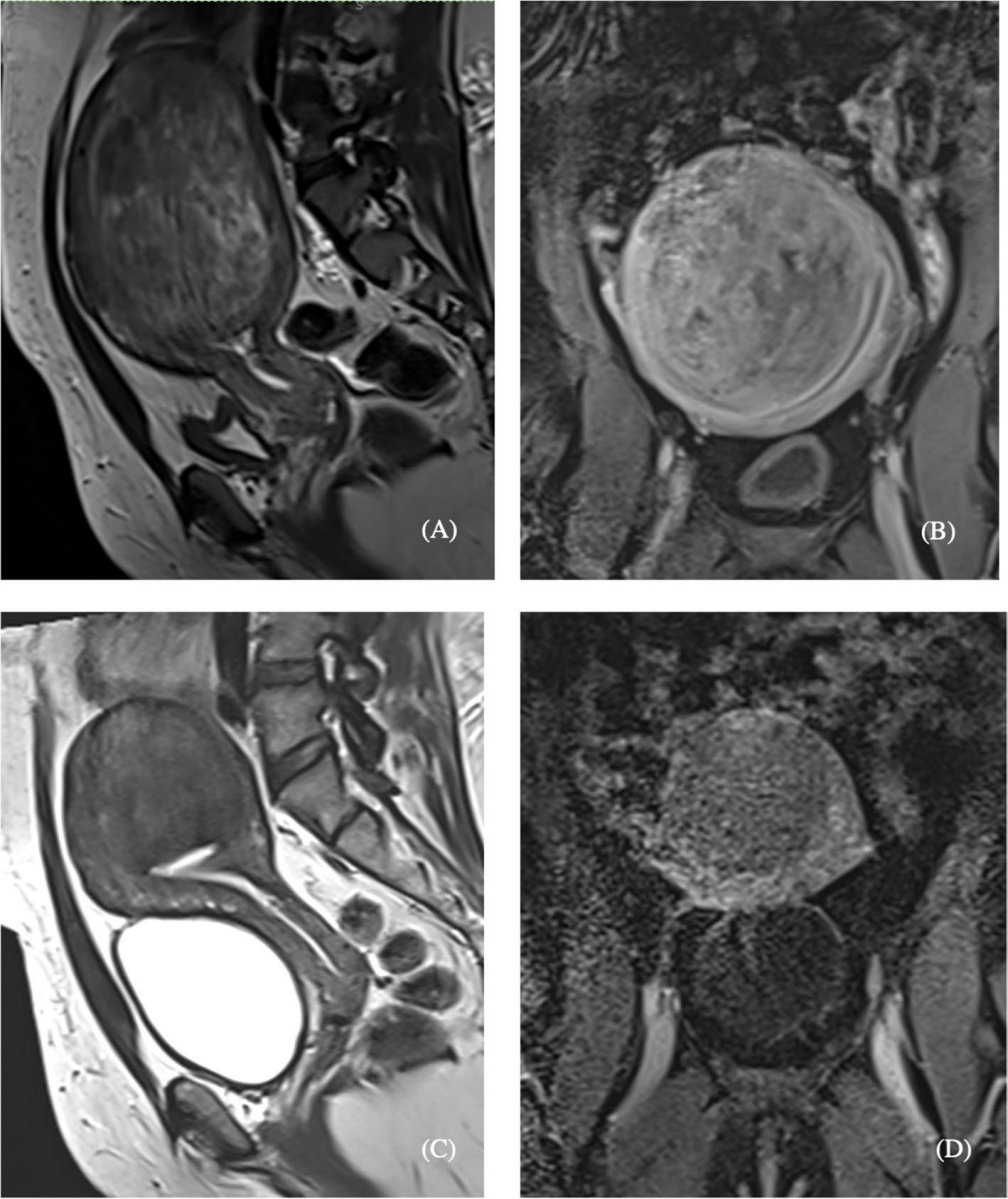
Pelvic MRI demonstrating a uterine fibroid measuring 12.3 × 7.2 × 11.5 cm (530 cm^3^) pre-procedure (**A** & **B**) and 6.8 × 6.0 × 7.6 cm (161 cm^3^) 6 months post-procedure (**C**&**D**). **A** & **C** Sagittal T2-weighted post-gadolinium. **B** & **D** Coronal T1-weighted post-gadolinium

**Fig. 5:**
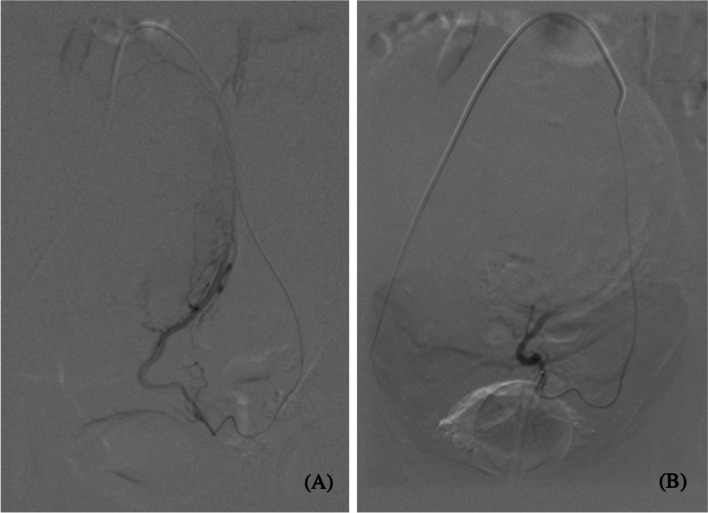
Selective left uterine artery digital-subtraction angiograms (30-degree left anterior oblique view) pre-embolization (**A**) and post-embolization (**B**). **A** demonstrates arterial blush and uterine artery branches linked with the fibroid. **B** demonstrates occlusion of flow of the vessel branches feeding the fibroid, absence of arterial blush, and patency of the left uterine artery

Overall, 100.0% (*n* = 38/38) of procedures were technically successful. There was no major complication and one minor complication (SIR Class A): post-embolization syndrome, which was managed conservatively with no long-term sequelae (Table 3). Self-reported symptom-severity scores at six-months post-procedure decreased in all categories: abnormal uterine bleeding from 8.8 to 3.1 (-5.7), pain from 6.7 to 3.2 (-3.5), and bulk symptoms from 2.8 to 1 (-1.8) (*p* < 0.001) (Fig. 1). 100% (*n* = 38/38) reported satisfaction with their symptom control. Pre-procedural dominant uterine fibroid volume and diameter were available for 52.6% (*n* = 20/38) of the study population with an average volume and maximum diameter of 155.6 cm^3^ and 7.0 cm, respectively. Of those patients, 6-month follow-up imaging was available from 45.0% (*n* = 9/20) of patients. On follow-up imaging, the average volume and diameter of the dominant fibroid decreased to 97.5 cm^3^ (-35.5%; *p* = 0.109) and 6.2 cm (-11.4%; *p* = 0.07), respectively (Figs. 2, 3, 4 and 5).

**Table 3:** Technical success and adverse events from uterine artery embolization in the study population

Variable	Characteristic	Frequency *n* = 38	Percent
Technical success	Yes	38	100.0
	No	0	0.0
Complications	No	37	97.4
	Yes^a^	1	2.6

^a^SIR class A, no therapy, no consequence

## Discussion

### Scope and impact

Black women bear the brunt of the fibroid burden globally, having a significantly higher prevalence and earlier onset compared to other racial groups [24, 25]. While research on the subject in SSA is severely limited, the impact of uterine fibroids is believed to be significant based on the racial composition of the region [6]. In a comprehensive scoping review on uterine fibroids in SSA, only five studies reported on the prevalence, each demonstrating significant variance across studies and populations [26]. Given the limited knowledge regarding fibroids in SSA, yet presumed high incidence and impact on the population, fibroids are a “silent epidemic” in the region.

As such, there has been minimal progress in improving patient outcomes in SSA in recent years. This is largely due to the limited treatment options available in the region, with hysterectomy and less commonly myomectomy typically being the only accessible options. While hysterectomy and myomectomy are effective treatment options, associated postoperative complications are not uncommon and include hemorrhage, infection, thromboembolic disease, adhesive disease, and organ damage [10, 27, 28]. In contrast, UAE presents an appealing alternative that may better align with patient preferences given its minimally invasive and uterine-preserving nature. While lamentably the vast majority of women in SSA currently do not have access to UAE, this study demonstrates that a UAE program is possible to implement in the region with our findings validating successful integration of UAE into clinical practice for the treatment of symptomatic fibroids and adenomyosis in Tanzania.

### Outcomes

The treatment of symptomatic fibroids with UAE is supported by strong evidence in the literature [29, 30]. Studies have repeatedly validated the safety and effectiveness of this procedure, demonstrating symptom control non-inferior to surgery, fewer major complications, and shorter hospitalization stays and recovery times [30–33]. Our experiences with UAE in Tanzania reflect the findings of prior studies. Adverse events and technical success rates in this study were on par with international standards at 2.6% and 97.3% compared to 4.4% and 95%, respectively [21, 30]. The mean volumetric response in this study (35.5%) was generally lower than previously reported [34]. However, the overall significance of this metric is controversial [34] and less than a quarter of patients in this study had both pre- and post-procedural imaging available for calculation. In contrast, patient satisfaction and symptom relief are considered the most important outcomes after UAE [34]. Patients in our cohort achieved adequate symptom control, including a significant reduction of self-reported symptom severity scores in all categories, as well as 100% reported satisfaction in symptom improvement. With high rates of technical and symptomatic success, and minimal complications observed in this study, it is evident that uterine artery embolization can safely and effectively be performed in a resource-limited setting. As such, improving access to this procedure in SSA should be prioritized as an evidence-based means to address the region’s high-burden of symptomatic fibroids.

### Uterine fibroid embolization awareness

Offering minimally invasive treatment options for symptomatic fibroids has far-reaching implications in a population at increased risk. In shared decision making with their treating physician, patients of different age groups, regions and religious beliefs are now afforded the opportunity to undergo UAE with qualified, locally-trained interventional radiologists in Tanzania. However, to date only a small fraction of women in the region that could potentially benefit from UAE, have been able to access the procedure.

Therefore, Igboeli et. al. proposes increased education and awareness as a means to bring attention to and address this public health crisis [6]. This includes improving knowledge of the different treatment options among clinicians and patients alike. Fostering shared decision making based on enhanced mutual understanding of the condition and available management options can effectively optimize patient outcomes and satisfaction. Following the introduction of UAE to the region, this is especially important, as the minimally invasive nature of this approach may better align with patient goals and priorities. Effective collaboration between specialties, as well as with patients themselves, is essential in providing optimal patient care, which has been an exhibited strength in the early experiences of Tanzania’s first UAE program. The hospital’s Department of Obstetrics and Gynecology (OB/GYN) provided most UAE referrals. The remaining patients were either self-referred or presented upon recommendation from relatives who had previously undergone the procedure. This demonstrates a promising trajectory of awareness with observed referral streams from both physicians specialized in the treatment of fibroids and satisfied patients by word-of-mouth. Due to the infancy of interventional radiology in SSA, it is important to continue to raise awareness among local clinicians and patients alike about the many benefits of procedures offered by the specialty [35]. This is especially important regarding symptomatic uterine fibroids, a condition in which women of color and those belonging to a lower socioeconomic bracket are often underdiagnosed and have a lower likelihood of receiving treatment [36] – an issue that can be mitigated through increased awareness and education.

### Clinical implications

UAE is not only an effective treatment for symptomatic fibroids and adenomyosis, but for postpartum hemorrhage as well, a common delivery complication and the leading cause of maternal mortality worldwide [37]. In SSA, lifetime risk of maternal mortality is estimated at 1 in 36 women [37], with 30–50% of maternal deaths attributed to PPH [38]. When conservative management fails, UAE is considered a first-line treatment option for patients and an evidence-based alternative to emergent hysterectomy [39].

However, given the relative novelty of interventional radiology at MNH, UAEs for PPH have not yet been integrated into clinical practice. Nonetheless, the service’s demonstrated proficiency in treating uterine fibroids and adenomyosis, reflected by high technical, clinical, and radiological success, suggest that the incorporation of UAE into management protocols for PPH would be practical. While the implementation of emergent UAEs for the treatment of PPH will take time in SSA, it will serve to benefit the patient population as it has done in high-income countries. Addressing this devastating and common obstetric emergency will be an important advancement for IR in the region, majorly impacting maternal health outcomes.

Developing a UAE program that includes emergent PPH, in tandem to elective fibroids and adenomyosis, has the potential to increase the long-term sustainability and accessibility of the program as a whole. Coverage by insurance and patients’ willingness to pay-out-of-pocket for elective procedures can generate enough revenue to subsidize emergent, life-saving UAEs. Striking a balance financially is important to ensure a self-sustaining operation capable of bridging inequities in health access and outcomes across the spectrum of procedural indications.

### Limitations

While the findings of this study demonstrate a promising proof-of-concept for the establishment of UAE programs in SSA, there are several limitations that should be considered. This is a single-center study and experiences implementing a UAE program may differ across the culturally, geopolitically, and socioeconomically diverse region. Additionally, given the novelty of UAE in Tanzania, the overall sample size of this study is relatively small and the safety and effectiveness of UAE in this setting for indications other than uterine fibroids, including adenomyosis and PPH, cannot be meaningfully interpreted and/or are conjectural. In addition, symptom impact on quality of life was not assessed and the evaluation of symptom severity was not performed using a validated measure. Finally, both baseline and follow-up imaging were only available for a subset of the study population due to MNH not having an established picture archiving and communication system, which introduced selection bias to all radiological analyses. Additional research is needed to further assess the safety and effectiveness of uterine artery embolization for different indications and across different populations and healthcare systems in sub-Saharan Africa.

## Conclusions

Uterine artery embolization is a procedure that well-exemplifies the potential role that IR can play in global health at large. While IR is still a specialty in its infancy in nations such as Tanzania, its rapid trajectory of growth in the region shows promise in how it may impact critical areas in resource-limited settings, including maternal and reproductive health. Less than 5 years old, the Tanzania IR training program has already produced ten graduates sufficiently trained in UAE, as demonstrated in this study, who have since spread out across Tanzania, as well as to Rwanda and Nigeria. With additional IR training programs launching in neighboring countries, Kenya in 2020 and Rwanda and Uganda in 2023, a domino effect is underway expanding the reach of the specialty in East Africa. Sustaining this growth is essential in order to ensure equitable access to IR services, including UAE. As uterine artery embolization has emerged as a standard of care option to manage symptomatic uterine fibroids and postpartum hemorrhage in high-resource settings, with adequate support and investment, the same is possible in SSA.

## Data Availability

Anonymized data not published within this article will be made available by the corresponding author, upon reasonable request. We take full responsibility for the data, analyses, interpretation and research conduct. We have full access to all data and have obtained the rights to publish these results.

## Abbreviations

IR: Interventional Radiology
UAE: Uterine Artery Embolization
SSA: Sub-Saharan Africa
MNH: Muhimbili National Hospital
PPH: Postpartum Hemorrhage
OB/GYN: Obstetrics and Gynecology
